# Supervised and Unsupervised Physical Activity Programs in Pregnancy—There Is a Benefit for Both

**DOI:** 10.1155/jdr/9981189

**Published:** 2026-05-19

**Authors:** Christina Sitzberger, Annette Wacker-Gußmann, Renate Oberhoffer, Silvia Lobmaier, Cody Strom, Samantha McDonald, Alex Claiborne, Linda May

**Affiliations:** ^1^ Department of Health and Sport Sciences, Applied Sport Sciences, TUM School of Medicine and Health, Munich, Germany; ^2^ Department of Congenital Heart Disease and Pediatric Cardiology, German Heart Center Munich, Technische Universität München, Munich, Germany, dhm.mhn.de; ^3^ Department of Health and Sport Sciences, Institute of Preventive Paediatrics, TUM School of Medicine and Health, Munich, Germany; ^4^ Department of Gynecology and Obstetrics, Klinikum Rechts der Isar, Technische Universität München, Munich, Germany, tum.de; ^5^ Kinesiology and Sports Department, University of Southern Indiana, Evansville, Indiana, USA, usi.edu; ^6^ School of Kinesiology and Recreation, Illinois State University, Normal, Illinois, USA, illinoisstate.edu; ^7^ Department of Kinesiology, Nutrition, and Health, Miami University, Oxford, Ohio, USA, miami.edu; ^8^ Department of Kinesiology, Human Performance Lab, East Carolina University, Greenville, North Carolina, USA, ecu.edu; ^9^ Department of Obstetrics and Gynecology, East Carolina University, Greenville, North Carolina, USA, ecu.edu

**Keywords:** birth outcome, body mass index, high-risk pregnancy, physical activity, pregnancy, sports program, supervised sports program

## Abstract

**Objectives:**

Regular physical activity (PA) during pregnancy reduces the risk of cardiometabolic disease. The World Health Organization recommends a minimum of 150 min/week of moderate‐intensity exercise for healthy pregnant women. However, these guidelines are not commonly met, and implications for pregnancy outcomes are not fully understood. This study is aimed at determining whether differences between a supervised and an independent training program affect gestational weight gain and birth outcomes.

**Methods:**

Four hundred and seventy‐eight pregnancies in two countries were investigated with either a supervised or unsupervised exercise program during pregnancy to compare maternal characteristics, PA levels, and birth outcomes and determine predictors of infant morphometrics.

**Results:**

Maternal participants had an average age of 31.7 years (±4.47) with a body mass index (BMI) of 25.07 (±5.18). Analysis of weight gain during pregnancy showed significant differences (*p* = 0.007), with a substantial increase in the supervised training program cohort (21.99 ± 36.34) compared to a moderate increase in the unsupervised cohort (14.22 ± 10.91). Women in the unsupervised group engaged in more intense and frequent physical activities. Weight gain during pregnancy had the most significant influence on birth weight. The most significant predictor of birth BMI was the intensity of maternal PA.

**Conclusions:**

Supervised sports programs are helpful but not necessary to influence pregnancy outcomes. The intensity of PA is the most important issue.

**Summary**

• Higher levels of physical activity during pregnancy are associated with reduced gestational weight gain (GWG).
• Both moderate and vigorous physical activities throughout pregnancy confer significant health benefits.
• Maternal exercise contributes to improved health outcomes for both the mother and the child.

## 1. Background

Engaging in regular physical activity during pregnancy reduces the risk of several complications, including gestational diabetes mellitus (GDM), hypertensive disorders, and excessive GWG [1]. The World Health Organization (WHO) recommends that pregnant women without contraindications engage in at least 150 min of moderate‐intensity aerobic physical activity each week to augment gestational health [2]. This recommendation aligns with guidelines from various health organizations worldwide, emphasizing the importance of staying active during pregnancy [3, 4].

The positive effects of physical activity extend beyond the mother to benefit the fetus. Regular physical activity during pregnancy is positively associated with birth weights in the normal range and improved fetal heart rate (HR) patterns, contributing to overall better fetal health outcomes [5]. Moreover, physical activity does not increase the risk of adverse outcomes such as stillbirth, miscarriage, or low birth weight, making it safe and beneficial for most pregnant women [6, 7]. This evidence helps dispel common misconceptions and fears that prenatal physical activity could harm the developing fetus. Thus, women should be encouraged to become or remain active during pregnancy.

Despite the well‐documented advantages of physical activity during pregnancy, many pregnant women do not meet the recommended levels [7]. Barriers such as uncertainty about safety, lack of guidance from healthcare providers, and physical discomfort during pregnancy contribute to lower activity levels [7].

Several studies show different results concerning the effects of supervised, controlled exercise programs versus independent exercise training programs. These studies showed that both types of exercise programs during pregnancy can provide health benefits. However, supervised exercise studies pose significant problems, including low retention rates and methodological issues such as geographical and sociological differences in populations and reliance on retrospective data [8–14].

The study is aimed at comparing selected parameters between supervised training program (SUP) and independent training program (IND) in pregnant women from two countries. These parameters focus on maternal characteristics, physical activity during pregnancy, birth outcomes of offspring, and predictors of morphometric outcomes in infants, such as body mass index. The hypothesis is that SUPs have greater health benefits than unsupervised programs.

## 2. Materials and Methods

Subjects from two comparable living regions, urban residential areas, in two countries (United States and Germany) were compared. The environments of the two regions offer pregnant women numerous opportunities to engage in physical activities in a healthy environment, as the regions lie in the continental climate zone with distinct seasons that offer everything from summer sun to winter snow.

Data focused on maternal characteristics, physical activity levels, offspring birth outcomes, and predictors of infant morphometric outcomes, such as body mass index.

Data from SUP and IND from two countries were compared. This involved two studies in the respective countries. The following section describes the methods used in each study and finally compares the datasets that can be compared.

The SUP dataset analyzed the impact of supervised exercise types (aerobic, resistance, and concurrent) during pregnancy on offspring health outcomes in a post hoc secondary analysis of a randomized control trial (RCT). The study compared these exercise modes to a control group with no exercise. Approved by the East Carolina University Institutional Review Board (#12‐002425) and following the Declaration of Helsinki, the study recruited pregnant women via fliers at local clinics between July 2015 and January 2018. After informed consent, women were enrolled between 13 and 16 weeks of gestation. All participants in the exercise groups followed a supervised program three times per week until delivery and completed the Modifiable Physical Activity Questionnaire (MPAQ) for prepregnancy physical activity. Sessions included a 5‐min warm‐up, 50 min of randomized activity, and a 5‐min cooldown. Aerobic activities, tailored to maintain moderate intensity (40%–59% HR reserve [HRR], Borg’s Rating of Perceived Exertion Scale [RPE] 12–14), included treadmill, bike, or elliptical exercises. Strength conditioning involved 15‐rep sets at moderate intensity (60% one repetition maximum [RM]) using various equipment. Concurrent training combined circuits lasting 4.5–5 min each. The control group completed 50‐min low‐intensity stretching and breathing sessions, monitored to remain below 40% HRR and RPE < 12. Eligible participants were healthy, low‐risk, nonsmoking women aged 18–40 years, with a prepregnancy BMI of 18.5–39.9 kg/m^2^, singleton pregnancies, and no contraindications to exercise. Exclusion criteria included smoking, pre‐existing diabetes, hypertension, cardiovascular disease, and conditions affecting fetal growth, such as systemic lupus erythematosus.

### 2.1. SUP Maternal and Birth Measures

Key maternal and infant data were collected from prescreening eligibility forms, postpartum questionnaires, and electronic health records. This included maternal age, gravida, parity, prepregnancy weight and height, GWG, length of gestation, infant sex, birth length, and birth weight. Exercise metrics (frequency, duration, and intensity, type) were tracked using training logs throughout pregnancy. GWG was calculated from the difference between prepregnancy and delivery weight, with prepregnancy weight collected via eligibility questionnaires. Physical activity data were assessed at 16 and 36 weeks using the MPAQ. Maternal height and weight were measured with a stadiometer and medical grade scale, and BMI was calculated as weight (kilogram)/height (square meter), categorized as healthy (18.5–24.9), overweight (25–29.9), or obese (> 30).

For the IND dataset, pregnant women were included in a prospective controlled observational study. They were assessed and recruited during their routine examination in the hospital. Pregnant women were recruited from the second trimester of pregnancy onwards. The inclusion criteria were as follows: pregnant women > 18 years old, good language skills, and no restrictions for any sporting activity. The participants could decide for themselves which physical activity they wanted to do. There was no supervised and controlled sports program within the study. Exclusion criteria were cardiovascular or nephropathic diseases, multiple pregnancies, acute illness, including infectious diseases, and premature labor.

The local ethics committee approved the study (464/15s). All participants gave their written informed consent.

The pregnant women were examined according to a predefined published protocol [15]. The protocol included general health data of the mother. Anthropometric data were also collected on children.

### 2.2. IND Maternal and Birth Measures

Data collected included maternal age, gravida, parity, prepregnancy weight and height, GWG, length of gestation, infant sex, birth length, and birth weight. Maternal physical activity before and during pregnancy was assessed using standardized tools, including an international physical activity questionnaire and a fitness test (6‐min walk test [16] based on American Thoracic Society guidelines [17]). MET values for activities were assigned using the compendium of physical activities [18]. The 6‐min walk test was conducted at the study’s start.

For both groups, anthropometric data of the mother and the children were standardized and collected in the same way for both groups from the maternity pass and the children’s examination booklet [19, 20]. The frequency (how often per week/month) and type of physical activity were derived from participant self‐reports in both groups via questionnaire. The SUP group reported the number of completed sessions according to the predefined training plan, while the IND group indicated the frequency and type of activities performed during pregnancy. The classification of activity intensity (low/moderate/vigorous) in METs was performed for both groups using the Compendium of Physical Activities, following the same standardized approach for both cohorts.

## 3. Statistical Analysis

Statistical analysis was conducted using SPSS Version 25. Continuous variables were reported as mean ± standard deviation and categorical variables as counts and percentages. The Mann–Whitney *U* test assessed differences between group means, while Pearson’s chi‐square test analyzed categorical variables. A multivariate logistic model examined the influence of age, height, prebirth weight, BMI, GWG, and METs (average and maximum before and during pregnancy) on the child’s birth weight and BMI to identify the strongest predictor. Statistical significance was set at *p* = 0.05. Subgroup analysis was performed.

## 4. Results

A total of 478 women were included in the study. The average age of pooled participants was 31.7 (±4.47) years, with a BMI of 25.07 (±5.18). The following tables contain missing values due to incomplete datasets from some participants and variations in the total number of participants, resulting in percentages that do not always sum to 100%. Maternal demographic and anthropometric data demonstrate significant differences in the pregnant populations in the two study groups (Table 1).

**Table 1:** Anthropometric data.

	Study group	Sample size or classification	*M* *e* *a* *n* ± *S* *D*(%)	*p*
Age (years)	SUP	274	30.86 ± 4.27	**< 0.001**
	IND	203	32.82 ± 4.50	
Height (cm)	SUP	274	165.25 cm ± 6.69	**0.022**
	IND	204	166.62 cm ± 6.08	
Smoking	SUP	Yes	0.7%	**< 0.001**
		No	99.3%	
	IND	Yes	5.1%	
		No	91.9%	
Prepregnancy weight (kg)	SUP	274	69.50 kg ± 13.33	0.361
	IND	202	68.22 kg ± 17.32	
Prepregnancy BMI (kg/m^2^)	SUP	274	25.47 ± 4.65	**0.047**
	IND	202	24.52 ± 5.79	
Delivery weight (kg)	SUP	193	81.40 kg ± 14.22	0.778
	IND	171	81.86 kg ± 16.76	
Gestational weight gain (kg)	SUP	192	21.99 kg ± 36.34	**0.007**
	IND	171	14.22 kg ± 10.91	

*Note:* Results are based on Kruskal–Wallis or Mann–Whitney *U* tests due to not normally distributed data. Significance level: 0.05. The bold values indicate a significant difference.

Abbreviation: BMI, body mass index.

The data on weight gain revealed a significant difference between the substantial increase observed in the SUP group and the comparatively moderate increase in women from the IND group (Table 1).

Although self‐reports indicate higher and more frequent physical activity among the SUP group, average PA before and during pregnancy indicates that women from the IND group engaged in more intense and more frequent physical activities (Table 2). This trend is also evident in the classifications of MET groups (Table 2).

**Table 2:** Physical activity before and during pregnancy and maximum METs.

	Study group	Sample size or classification	Mean value (%)	*p*
Prepregnancy PA	SUP	Yes	75.2%	**< 0.001**
		No	15%	
	IND	Yes	67.2%	
		No	32.4%	
Prepregnancy PA frequency	SUP	1–2/week	74.1%	**< 0.001**
		1–2/month	1.1%	
		Never	15%	
	IND	1–2/week	54.5%	
		1–2/month	12.9%	
		Never	29.7%	
Prepregnancy average METs	SUP	204	4.30 ± 1.22	**< 0.001**
	IND	138	5.76 ± 1.21	
Prepregnancy average MET group classification	SUP	Light < 3	13.1%	**< 0.001**
		Moderate 3–6	55.5%	
		Vigorous > 6	6.6%	
	IND	Light < 3	2.9%	
		Moderate 3–6	40.7%	
		Vigorous > 6	23.5%	
Pregnancy PA	SUP	Yes	53.6%	**< 0.001**
		No	0.7%	
	IND	Yes	51%	
		No	37.3%	
Pregnancy PA frequency	SUP	1–2/week	53.1%	**< 0.001**
		1–2/month	0.7%	
		Never	1.1%	
	IND	1–2/week	37.7%	
		1–2/month	14.2%	
		Never	33.8%	
Pregnancy average METs	SUP	269	3.37 ± 0.91	**< 0.001**
	IND	114	4.76 ± 1.43	
Pregnancy average MET group classification	SUP	Light < 3	36.1%	**0.003**
		Moderate 3–6	59.9%	
		Vigorous > 6	4.1%	
	IND	Light < 3	22.8%	
		Moderate 3–6	65.8%	
		Vigorous > 6	11.4%	
Prepregnancy max METs	SUP	205	5.87 ± 1.97	**< 0.001**
	IND	138	6.54 ± 1.30	
Pregnancy max METs	SUP	268	4.41 ± 1.88	**< 0.001**
	IND	114	5.35 ± 1.69	
Prepregnancy max MET group classification	SUP	Light < 3	10.1%	**< 0.001**
		Moderate 3–6	32.1%	
		Vigorous > 6–9	35%	
	IND	Light < 3	2%	
		Moderate 3–6	25%	
		Vigorous > 6–9	40.7%	
Pregnancy max MET group classification	SUP	Light < 3	48%	**< 0.001**
		Moderate 3–6	33.8%	
		Vigorous > 6–9	18.2%	
	IND	Light < 3	27.2%	
		Moderate 3–6	52.3%	
		Vigorous > 6–9	20.4%	

*Note:* Results are based on Kruskal–Wallis or Mann–Whitney *U* tests due to not normally distributed data and chi‐squared coefficient. Significance level: 0.05. The bold values indicate a significant difference.

Abbreviations: MET, metabolic equivalent; PA, physical activity.

An assessment of the maximum METs performed before and during pregnancy noted a significant difference between the two groups of pregnant women (*p* value Table 2). Both before and during pregnancy, the women from the IND group trained with higher MET values than those from the SUP group (*p* value Table 2). Accordingly, this can also be seen in the classification of the METs groups, with more women from the IND group in the group of vigorous METs before and during pregnancy (*p* value Table 2).

Health‐related data of the children has a similar distribution of girls and boys: SUP group with 49.9% girls and 50.1% boys and IND group with 50.6% girls and 49.4% boys. Despite a similar distribution of males and females, the infants of the SUP group have different anthropometric measures relative to the IND group infants (*p* value Table 3). The children were also categorized according to whether they were born within the healthy weight range of 2500–4000 g [21].

**Table 3:** Health‐related data in the offspring.

	Study group	Sample size	Mean value (%)	*p*
Birth weight (kg)	SUP	272	3.45 kg ± 0.55	0.450
	IND	172	3.41 kg ± 0.40	
Weight within the healthy range^a^	SUP	272	Yes: 82.7%	**0.007**
			No: 17.3%	
	IND	172	Yes: 91.9%	
			No: 8.1%	
Birth weight *z*‐score	SUP	273	0.01 ± 1.34	0.123
	IND	79	‐0.14 ± 1.13	
Birth weight percentile	SUP	264	49.86 ± 28.03	0.094
	IND	79	45.48 ± 25.33	
Birth length (m)	SUP	271	0.49 m ± 0.02	**< 0.001**
	IND	79	0.52 m ± 0.02	
Birth length *z*‐score	SUP	273	−0.44 ± 2.47	**0.03**
	IND	79	0.39 ± 2.04	
Weight/length	SUP	271	6.95 ± 0.95	**< 0.001**
	IND	79	6.50 ± 0.64	
Weight/length *z*‐score	SUP	273	0.15 ± 1.48	**< 0.001**
	IND	79	−0.43 ± 1.15	
BMI (kg/m^2^)	SUP	271	14.05 ± 1.86	**< 0.001**
	IND	79	12.41 ± 1.29	
BMI *z*‐score	SUP	273	0.30 ± 1.40	**< 0.001**
	IND	79	−0.66 ± 1.08	
PI (kg/m^3^)	SUP	271	28.51 ± 4.34	**< 0.001**
	IND	79	23.78 ± 3.10	
PI *z*‐score	SUP	273	0.37 ± 1.25	**< 0.001**
	IND	79	−0.74 ± 0.94	
HC (mm)	SUP	255	0.34 mm ± 0.01	0.449
	IND	82	0.34 mm ± 0.01	
HC *z*‐score	SUP	269	−1.25 ± 5.77	0.306
	IND	78	−2.00 ± 6.43	

*Note:* Results are based on Kruskal–Wallis or Mann–Whitney *U* tests due to not normally distributed data. Significance level: 0.05. The bold values indicate a significant difference.

Abbreviations: BL, birth length; BMI, body mass index; HC, head circumference; PI, ponderal index; WtL, waist to length.

^a^Healthy range (2500–4000 g).

A comparison of the women categorized as “at risk” for GDM and/or hypertensive disorders of pregnancy (HDP) shows differences between women from the two countries in all variables (Table 4).

**Table 4:** Pregnant women “at risk”.

	Study group	Sample size or classification	Mean value (%)	*p*
At risk	SUP	Yes	50.7%	**< 0.001**
		No	49.3%	
	IND	Yes	70.6%	
		No	29.4%	
Age (years)	SUP	139	30.60 ± 4.151	**< 0.001**
	IND	143	32.92 ± 4.47	
Height (cm)	SUP	139	164.60 cm ± 6.70	**0.002**
	IND	144	167.12 cm ± 6.57	
Prepregnancy weight (kg)	SUP	139	77.31 kg ± 13.49	**0.004**
	IND	142	71.60 kg ± 19.23	
BMI (kg/m^2^)	SUP	139	28.66 ± 4.39	**< 0.001**
	IND	142	25.71 ± 6.39	
Gestational weight gain (kg)	SUP	86	21.86 kg ± 39.73	**0.035**
	IND	121	13.70 kg ± 12.25	
Prepregnancy average METs	SUP	103	4.36 ± 1.35	**< 0.001**
	IND	89	5.61 ± 1.18	
Pregnancy average METs	SUP	137	3.37 ± 0.93	**< 0.001**
	IND	78	4.86 ± 1.33	
Prepregnancy max METs	SUP	104	5.73 ± 1.96	**0.007**
	IND	89	6.40 ± 1.27	
Pregnancy max METs	SUP	137	4.47 ± 0.93	**0.006**
	IND	78	4.68 ± 1.33	

*Note:* MET index. “At risk”: overweight, GDM, thyroid disease. Results are based on Kruskal–Wallis or Mann–Whitney *U* tests due to not normally distributed data. Significance level: 0.05. The bold values indicate a significant difference.

Abbreviations: BMI, body mass index; MET, metabolic equivalent.

### 4.1. Regression Analysis

Multiple regression analysis was used to determine the strongest predictors influencing the weight and BMI of the children within the entire study group. The mother’s weight at delivery had the greatest influence on the child’s weight at birth (*β*: 0.53; *p* < 0.001; 95% CI [0.010, 0.025]). The same predictor applied to the birth weight *z*‐score (*β*: 0.526; *p* < 0.001; 95% CI [0.022, 0.062]) and the birth weight percentile (*β*: 0.579; *p* < 0.001; 95% CI [0.567, 0.1453]). The strongest predictor of the child’s BMI at birth was the average METs during pregnancy (*β*: −0.455; *p* < 0.001; 95% CI [−0.909, −0.282]). This also applied to the BMI *z*‐score (*β*: −0.423; *p* < 0.001; 95% CI [−0.706, −0.186]).

## 5. Discussion

The main findings of the study are that women in the unsupervised group engaged in more intense and frequent physical activities. The greatest predictor of neonatal BMI within the percentiles is the average METs during pregnancy, emphasizing the importance of achieving the recommended intensity of physical activity. Whether these METS are achieved by supervised or unsupervised training is not essential. Maternal GWG had the greatest influence on offspring weight at birth.

Recommended healthy levels of GWG are published by the *National Academy of Medicine* (*formerly the Institute of Medicine* [*IOM*]) guidelines, and excessive weight gain poses risks for both mother and child [22, 23]. For instance, high GWG increases the risk of pre‐eclampsia during pregnancy and is a significant predictor of long‐term overweight and obesity [24]. Similarly, GWG correlates positively with the birth weight of the child, regardless of the mother’s initial weight [25, 26].

Various epidemiological studies, including those conducted in Germany, have shown that excessive GWG increases the risk of macrosomia by two to three times [25–27]. A systematic review of the literature on the relationship between birth weight and the risk of overweight in later life found a positive linear relationship in 89% of the studies.

Our study shows that mothers and children from the SUP cohort have a significantly higher BMI and less PA than women and children from the IND cohort. These differences lead to a higher risk of overweight and obesity in children up to 4 years postbirth [28]. Additionally, the effects identified at birth can persist into adulthood, contributing to earlier and more severe metabolic disorders [28]. Hopkins et al. confirmed in their study that a significant reduction in birth weight among first‐time mothers, compared to a control group, contributes to a long‐term reduction in obesity rates [29].

In our study, the SUP cohort demonstrated a significantly higher frequency of physical activity both before and during pregnancy. The latter can be attributed to the study design, in which the intervention group in the SUP cohort was engaged in a structured exercise program, whereas pregnant women in the IND cohort chose their physical activities. In consequence, a higher percentage of women from the SUP cohort were physically active one to two times per week during pregnancy compared to women from the IND cohort. However, when examining the mean values of METs (metabolic equivalents) achieved before and during pregnancy, as well as the maximum METs achieved, the SUP cohort had less intensity of PA relative to the IND cohort. This finding is consistent with the classification of METs into light, moderate, and vigorous groups, revealing that significantly more women in the higher intensity categories were women of the IND cohort. This increased training volume during pregnancy is again associated with improved birth outcomes [28].

Our data, like other studies, show that neonatal BMI is linked to maternal exercise, particularly its intensity. Zavrosky et al. found that at least 16 MET hours per week and exercise intensity ≥ 60*%* of HR during pregnancy reduce the risk of hypertensive disorders and prevent excessive weight gain [30–33]. Maternal exercise dose has been associated with improved birth outcomes across the United States and other populations. While the impact of exercise intensity remains debated, moderate intensity (3–6 METs) is effective for health improvement and is recommended for beginners or unfit women, with gradual increases in activity. Short bursts of intense activity can provide similar benefits to longer moderate sessions, but maternal and fetal health should be carefully monitored [31].

There are many strengths to this unique analysis. This is the first comparison of pregnant women from two diverse populations. We additionally have a large sample of women with diverse BMIs as well as healthy and at‐risk pregnancies. However, there are also limitations. The weakness of the study is the nonuniform recruitment of both groups and the differences in the study design regarding the physical activity performed. The IND group was therefore not randomized, but all women who wanted to participate were included in accordance with the inclusion criteria. Although both studies utilized self‐reporting for prepregnancy PA, the IND cohort also used a self‐report during pregnancy, while the SUP cohort used a supervised exercise intervention, *potential reporting bias cannot be ruled out*. This could have led to inherent differences in the population. However, the allocation of METs for both groups was based on the standardized allocation via the compendium of physical activities [18]. This ensured that there were no differences in categorization. This made it possible to reliably classify the actual METs and thus determine the effort in both groups and categories them as low, moderate, or vigorous. The sociodemographic parameters, diet, and prenatal health behaviors were not analyzed separately and were not adjusted for the analysis.

Another limitation could be unmeasured regional differences that might have influenced the results. Further research is required to clarify these potential effects. The same applies to factors that were not measured, such as environmental conditions in the respective regions.

Overall, we were able to show that more intensive physical activity during pregnancy has a more positive effect on less weight gain during pregnancy. In addition, we showed that maternal exercise intensity is related to positive birth outcomes. Our study demonstrates that both approaches (supervised or unsupervised training) have beneficial effects. Supervised training has the advantage of promoting more consistent exercise participation throughout the week and pregnancy. However, independent training with an adequate metabolic equivalent of task also positively influences the health of both the mothers and their children. The key determinant in this context is the intensity of physical activity, measured in METs. These findings may serve as a bridge to encourage pregnant women, regardless of their individual exercise preferences, to engage in regular physical activity, provided obstetricians identify any contraindication.

Further studies are needed to determine whether the continuous implementation of physical activity during pregnancy leads to women continuing with physical activity in the long term after giving birth. This would improve health for both mother and child. In conclusion, both supervised and unsupervised physical activity will improve the health of women and children. There is an urgent need to establish the benefits of moderate and vigorous physical activity throughout pregnancy.

**Nomenclature**
BL: birth length
BMI: body mass index
GDM: gestational diabetes mellitus
GWG: gestational weight gain
HC: head circumference
HR: heart rate
HRR: reserve heart rate
INDs: independent training programs
IOM: National Academy of Medicine former Institute of Medicine
kg: kilogram
MET: metabolic equivalent
min: minute
MPAQ: Modifiable Physical Activity Questionnaire
PA: physical activity
PI: ponderal index
RCT: randomized control trial
RM: repetition maximum
RPE: Borg’s Rating of Perceived Exertion Scale
SUPs: supervised training programs
WHO: World Health Organization
WtL: waist to length

## Author Contributions

Conceptualization: C.S., A.W.‐G., and L.M. Methodology: C.S. and L.M. Software: C.S. and L.M. Data curation: C.S., A.W.‐G., L.M., Co.S., S.M., and A.C. Writing—original draft preparation: C.S., A.W.‐G., and L.M. Writing—review and editing: C.S., A.W.‐G., R.O., S.L., Co.S., S.M., A.C., and L.M. Supervision: A.W.‐G. Project administration: C.S., A.W.‐G., and L.M. Funding acquisition: L.M. and A.W.‐G. C.S. and A.W.‐G. have contributed equally to the manuscript.

## Funding

The study was funded by TUM‐IAS (PI Wacker‐Gussmann) and AHA (#15GRNT24470029, #18IPA34150006, #15GRNT24470029, and 18IPA34150006 [PI: May]) and Grant Numbers 24470029 and 34150006. Open Access funding was enabled and organized by Projekt DEAL.

## Disclosure

All authors have read and agreed to the published version of the manuscript.

## Ethics Statement

The study was approved by the East Carolina University Institutional Review Board (#12‐002425). It was conducted according to the guidelines of the Declaration of Helsinki and approved by the Institutional Review Board (or Ethics Committee) of TUM University (464/15s).

## Consent

Informed consent was obtained from all subjects involved in the study. Written informed consent has been obtained from the patient(s) to publish this paper.

## Conflicts of Interest

The authors declare no conflicts of interest.

## Data Availability

The datasets generated and/or analyzed during the current study are not publicly available due to sensitive information from the participants but are available from the corresponding author on reasonable request.
